# U.S. medical organizations and climate change advocacy: a review of public facing websites

**DOI:** 10.1186/s12889-022-14339-7

**Published:** 2022-10-21

**Authors:** Thomas Bush, William A. Jensen, Tamiko R. Katsumoto

**Affiliations:** 1 Department of Medicine, Santa Clara Valley Medical Center, San Jose, CA USA; 2 Department of Medicine (Retired), Santa Clara Valley Medical Center, San Jose, CA USA; 3 Department of Medicine, Division of Immunology and Rheumatology, Stanford University School of Medicine, Stanford, Palo Alto, CA USA

**Keywords:** Climate change, Global warming, Greenhouse, Carbon footprint, Mitigation, Adaptation, Green practice, Medical organizations, Climate emergency

## Abstract

**Background:**

Climate change poses a risk of health catastrophes and must be expeditiously addressed across the health care sector. Physicians are considered trustworthy and are well positioned to discuss climate change with patients. A unified strategy by all U.S. medical societies is essential to effectively mitigate their carbon footprint and address health concerns.

**Methods:**

We conducted a review of the public facing websites of member organizations of the AMA House of Delegates and the AMA, which were scored based on inclusion of content related to climate change in position statements or policies, task forces or committees, patient education materials, practice recommendations and any official society publications. Membership in the Medical Society Consortium on Climate and Health or participation in the organization My Green Doctor were recorded as indicators of a commitment to providing educational resources about mitigation and adaptation to climate change. The availability of a virtual option for annual meetings, as a potential means to reduce the carbon footprint of attendees, was trended from 2021 to 2022.

**Results:**

Fifty out of 111 U.S. medical organizations (45%) had at least one metric with a reference to climate change and sixty-one organizations (55%) had no evidence of such website content. Out of 111 websites, only 20% (*N* = 22) had position statements or policies pertaining to climate change, 11% (*N* = 12) had committees or task forces dealing with climate change, 8% (*N* = 9) provided patient education resources on climate change, 21% (*N* = 23) included green practice recommendations and 45% (*N* = 50) had an article in an official society publication addressing climate change. Only 14% (*N* = 15) were listed as member societies of the Medical Consortium on Climate Change and 2% (*N* = 2) were participating organizations with My Green Doctor.

**Conclusions:**

Viewed through the lens of medical society websites, there was a wide variation in efforts to address climate change. The high performing organizations can serve as a guide for other societies to help mitigate and adapt to the climate emergency.

**Supplementary Information:**

The online version contains supplementary material available at 10.1186/s12889-022-14339-7.

## Background

Excess generationof greenhouse gasses (GHG) has resulted in profound changes in the earth’s climate, with a risk of widespread health catastrophes related to extreme weather events, heat waves, drought, increased wildfires and a spread in vector-borne diseases [1–6]. Medical experts have demanded that our political leaders increase efforts to reduce carbon emissions [7]. The U.S. health sector also needs to closely examine its own practices, since it accounts for nearly 8% of the country’s CO_2_ emissions [8] and is responsible for the well-being of the U.S. public.

The health care sector must achieve a number of goals to substantially decarbonize and to address the health of their patients: 1) it must work to reduce the industry's carbon footprint and develop a sustainable business model both for medical centers and individual practices, 2) it must foster research to better understand the myriad of health implications of climate change for susceptible patients, and 3) it must educate physicians and patients regarding appropriate steps in mitigation and adaptation [9–11].

A potential strategy to achieve these goals to combat the climate emergency is to leverage our medical societies [12] that represent the majority of physicians [13] who together care for virtually the entire population of the U.S. Maibach et al. have called on medical societies to develop strong climate and health resolutions and policies and to advocate for them at both the state and federal levels [14]. As a united front, medical societies could be an influential force to catalyze changes in our current practices to reduce the health sector's carbon footprint. Moreover, medical societies can shape the discussion about climate change with physicians and their patients by providing increased education on the health effects of climate change and how patients themselves can help mitigate climate change. Physicians can leverage their status as one of the most trustworthy sources of information regarding climate change and their perceived responsibility to educate patients and policymakers about its health impacts [15, 16].

The objective of this study was to assess the current activities of U.S. medical societies to reduce their carbon footprint and address the health risks that the climate emergency poses for their patients. Their websites offer an opportunity to examine organizational initiatives regarding their climate change activities [17, 18]. Our focus was to assess the content of U.S. medical society websites related to climate change and, as such, we developed a novel set of metrics. We hope that the information presented will encourage organizations and their members to critically examine and improve upon their current efforts.

## Methods

### Study design

In this observational study, we analyzed the websites of U.S. Medical Organizations and extracted data between March 5–22, 2022.

### Sample selection

U.S. medical organizations were selected for inclusion based on a list of member national medical specialty societies of the AMA House of Delegates, which was utilized to ensure a broad representation [19]. Two authors (TB and WJ) independently used a predesigned form to carry out data extraction from websites of the member organizations of the AMA House of Delegates. The search strategy and a weighted score of climate change parameters was developed de novo based on characteristics deemed to be most relevant for assessing an organization’s commitment to climate change. In our review of the literature, we were unable to find prior examples of such metrics. Each organization's website was systematically screened for climate change content by evaluating position statements, policies, committees and task forces, patient education materials, practice recommendations, and any official society publications or blogs. Key word searches were performed using “climate change”, “global warming”, “green practice” and “carbon footprint”. Organizations with websites that lacked a functional search box were excluded, as this was a key tool in identifying relevant website content (Additional File 1). Disagreements regarding content were resolved by reaching consensus with a third author (TK).

Some medical societies partner with environmentally sustainable health care organizations, and this was considered an important indicator of the medical society’s commitment to provide members with educational resources about mitigation and adaptation to climate change. There are a number of climate focused organizations that serve as valuable resources for medical societies. A review of several prominent sustainable health care organizations’ websites, including Climate and Health Alliance, Healthcare without Harm, Practice Greenhealth and Planetary Health Alliance [20–23], produced two organizations that specifically referenced the participation of U.S. medical societies: 1) the Medical Society Consortium on Climate and Health [24] and 2) My Green Doctor [25]. Their websites noted a number of member organizations, but we only included those on the list of national medical specialty societies of the AMA House of Delegates as a metric for this study.

### Analysis

We developed a weighted score based on the relative importance of different domains evaluated: 4 points were assigned for a) inclusion of climate change issues in a position statement or policy, b) a climate change focus in a task force or committee, and c) membership in the Medical Society Consortium on Climate and Health or My Green Doctor [24, 25]. Two points were assigned for a) patient education regarding climate change and b) green practice recommendations. One point was assigned for articles regarding climate change in official society publications or blogs. The presence of climate change content in position statements, policies, task forces and committees was given higher weighting than the provision of patient education materials or practice recommendations, as they were felt to provide more strategic direction to the organizational efforts. Articles in official society publications or blogs were scored lower since their inclusion was not necessarily controlled by the societies. The maximum obtainable score was 21 points (100%).

In addition to the climate change topics covered by the scored metrics, we also examined the format of the medical societies’ annual meetings, as a virtual option for attendance in the future could be leveraged to reduce an organizations’ carbon footprints [26]. The attendance format for a sample of 2021 and 2022 annual meetings, primarily driven by concerns about the COVID-19 pandemic, was recorded as virtual only, a hybrid of virtual and in-person, or in-person only. This data is exploratory and was not scored in this survey, however in future surveys, the offering of a hybrid option could be considered a scored element as a carbon reduction strategy.

## Results

**Fig. 1:**
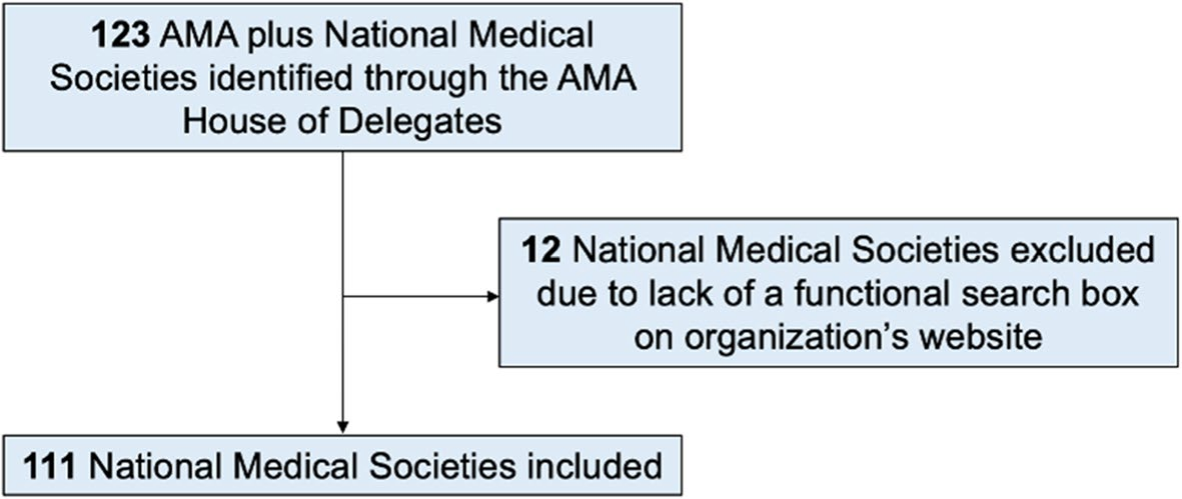
Sample selection criteria for organizations

We identified a total of 123 medical societies based on the AMA House of Delegates listed on the AMA website. Of those, 12 websites lacked a functional search box and were excluded from the review (Fig. 1 and Additional File 1).

We searched each of the 111 organizations’ websites for 5 metrics regarding climate change (position statements, committees, patient education, green practice recommendations and publications), for a total of 555 data points (Table 1). The results for the Medical Society Consortium on Climate and Health and My Green Doctor were abstracted directly from those organizations’ websites. There were 35 discrepancies (6%) between the 2 reviewers on their initial search of the 555 data points, all of which were resolved upon further review. The website sections on committees, policies or position statements were found to be password protected on 11 of the 111 websites. This limited access for a complete website review for these organizations, as indicated in Table 1. Blocked data was still discoverable on some of these websites due to overlapping information sources: as an example, although access to a committee list was protected, an accessible climate change position statement referred to the existence of a relevant climate change committee. Obtaining information regarding the content of official society publications or the format of annual meetings often required a search of the internet outside of the organizations’ websites.

**Table 1:** Climate Change Scoring Metrics for National Medical Organizations

National Medical Organization	Total score (% maximum)	Position statement or policy (a)	Committee or task force (a)	Patient education (b)	Practice recommend-ations (b)	Publication or blog (c)	Medical Society Consortium (d)	My Green Doctor (d)
American Academy of Family Physicians	19 (90)	4	4	2	0	1	4	4
American Academy of Pediatrics	17 (81)	4	4	2	2	1	4	0
American College of Physicians	17 (81)	4	4	2	2	1	4	0
American Medical Association	17 (81)	4	4	2	2	1	4	0
Infectious Disease Society of America	17 (81)	4	4	2	2	1	4	0
American Academy of Allergy Asthma and Immunology	15 (71)	4	4	2	0	1	4	0
American Academy of Dermatology (e)	15 (71)	4	0	0	2	1	4	4
American Psychiatric Association	15 (71)	4	4	2	0	1	4	0
American College of Obstetricians and Gynecologists	13 (62)	4	4	0	0	1	4	0
American Thoracic Society	13 (62)	4	4	2	2	1	0	0
American Academy of Ophthalmology	11 (52)	4	0	0	2	1	4	0
American Academy of Orthopaedic Surgeons (e)	11 (52)	4	4	0	2	1	0	0
American Academy of Physical Medicine and Rehabilitation	11 (52)	4	0	0	2	1	4	0
American College of Occupational and Environmental Medicine	11 (52)	4	0	0	2	1	4	0
American Society of Anesthesiologists (e)	11 (52)	4	4	0	2	1	0	0
American College of Emergency Physicians	9 (43)	4	0	0	0	1	4	0
American College of Preventive Medicine	9 (43)	4	0	0	0	1	4	0
American College of Radiology	9 (43)	4	4	0	0	1	0	0
American Society for Clinical Pathology	7 (33)	4	0	0	2	1	0	0
The Endocrine Society	7 (33)	4	0	0	2	1	0	0
American Academy of Child and Adolescent Psychiatry (e)	5 (24)	0	0	2	2	1	0	0
American Academy of Neurology	5 (24)	4	0	0	0	1	0	0
American College of Cardiology	5 (24)	4	0	0	0	1	0	0
American Geriatrics Society	5 (24)	0	0	0	0	1	4	0
American Association of Public Health Physicians	3 (14)	0	0	0	2	1	0	0
American College of Gastroenterology (e)	3 (14)	0	0	0	2	1	0	0
American College of Surgeons	3 (14)	0	0	0	2	1	0	0
American Gastroenterological Association	3 (14)	0	0	0	2	1	0	0
American Society for Gastrointestinal Endoscopy	3 (14)	0	0	0	2	1	0	0
American Society for Reproductive Medicine	3 (14)	0	0	0	2	1	0	0
American Society of Cataract and Refractive Surgery	3 (14)	0	0	0	2	1	0	0
Radiological Society of North America	3 (14)	0	0	0	2	1	0	0
Society of Hospital Medicine	3 (14)	0	0	0	2	1	0	0
American Association for Hand Surgery	1 (5)	0	0	0	0	1	0	0
American Association of Neurological Surgeons	1 (5)	0	0	0	0	1	0	0
American Association of Plastic Surgeons	1 (5)	0	0	0	0	1	0	0
American College of Allergy Asthma Immunology (e)	1 (5)	0	0	0	0	1	0	0
American College of Chest Physicians (CHEST)	1 (5)	0	0	0	0	1	0	0
American College of Rheumatology	1 (5)	0	0	0	0	1	0	0
American Orthopaedic Association	1 (5)	0	0	0	0	1	0	0
American Rhinologic Society	1 (5)	0	0	0	0	1	0	0
American Society for Radiation Oncology	1 (5)	0	0	0	0	1	0	0
American Society of Hematology	1 (5)	0	0	0	0	1	0	0
American Society of Neuroradiology	1 (5)	0	0	0	0	1	0	0
American Society of Plastic Surgeons (e)	1 (5)	0	0	0	0	1	0	0
American Urological Association	1 (5)	0	0	0	0	1	0	0
Association for Clinical Oncology	1 (5)	0	0	0	0	1	0	0
Association of University Radiologists	1 (5)	0	0	0	0	1	0	0
Renal Physicians Association	1 (5)	0	0	0	0	1	0	0
Society for Vascular Surgery	1 (5)	0	0	0	0	1	0	0
Academy of Physicians in Clinical Research	0	0	0	0	0	0	0	0
Aerospace Medical Association	0	0	0	0	0	0	0	0
AMDA—The Society for Post-acute and Long-term Care Medicine	0	0	0	0	0	0	0	0
American Academy of Cosmetic Surgery	0	0	0	0	0	0	0	0
American Academy of Facial Plastic and Reconstructive Surgery	0	0	0	0	0	0	0	0
American Academy of Hospice and Palliative Medicine	0	0	0	0	0	0	0	0
American Academy of Insurance Medicine (e)	0	0	0	0	0	0	0	0
American Academy of Otolaryngic Allergy Inc	0	0	0	0	0	0	0	0
American Academy of Otolaryngology -Head and Neck Surgery	0	0	0	0	0	0	0	0
American Academy of Sleep Medicine	0	0	0	0	0	0	0	0
American Association for Thoracic Surgery	0	0	0	0	0	0	0	0
American Association of Clinical Endocrinology	0	0	0	0	0	0	0	0
American Association of Gynecologic Laparoscopists	0	0	0	0	0	0	0	0
American Association of Neuromuscular & Electrodiagnostic Medicine	0	0	0	0	0	0	0	0
American Clinical Neurophysiology Society (e)	0	0	0	0	0	0	0	0
American College of Medical Genetics and Genomics	0	0	0	0	0	0	0	0
American College of Medical Quality	0	0	0	0	0	0	0	0
American College of Mohs Surgery (e)	0	0	0	0	0	0	0	0
American College of Nuclear Medicine	0	0	0	0	0	0	0	0
American College of Radiation Oncology	0	0	0	0	0	0	0	0
American Institute of Ultrasound in Medicine	0	0	0	0	0	0	0	0
American Orthopaedic Foot and Ankle Society	0	0	0	0	0	0	0	0
American Roentgen Ray Society	0	0	0	0	0	0	0	0
American Society for Dermatologic Surgery (e)	0	0	0	0	0	0	0	0
American Society for Metabolic and Bariatric Surgery	0	0	0	0	0	0	0	0
American Society for Surgery of the Hand	0	0	0	0	0	0	0	0
American Society of Addiction Medicine	0	0	0	0	0	0	0	0
American Society of Breast Surgeons	0	0	0	0	0	0	0	0
American Society of Colon and Rectal Surgeons	0	0	0	0	0	0	0	0
American Society of Cytopathology	0	0	0	0	0	0	0	0
American Society of Dermatopathology	0	0	0	0	0	0	0	0
American Society of General Surgeons	0	0	0	0	0	0	0	0
American Society of Interventional Pain Physicians	0	0	0	0	0	0	0	0
American Society of Maxillofacial Surgeons	0	0	0	0	0	0	0	0
American Society of Nuclear Cardiology	0	0	0	0	0	0	0	0
American Society of Ophthalmic Plastic and Reconstructive Surgery	0	0	0	0	0	0	0	0
American Society of Retina Specialists	0	0	0	0	0	0	0	0
American Society of Transplant Surgeons	0	0	0	0	0	0	0	0
American Vein and Lymphatic Society	0	0	0	0	0	0	0	0
AMSUS The Society of Federal Health Professionals	0	0	0	0	0	0	0	0
College of American Pathologists	0	0	0	0	0	0	0	0
Congress of Neurological Surgeons	0	0	0	0	0	0	0	0
Contact Lens Association of Ophthalmologists	0	0	0	0	0	0	0	0
Heart Rhythm Society	0	0	0	0	0	0	0	0
International Society for the Advancement of Spine Surgery	0	0	0	0	0	0	0	0
International Society of Hair Restoration Surgery	0	0	0	0	0	0	0	0
National Association of Medical Examiners	0	0	0	0	0	0	0	0
North American Neuromodulation Society	0	0	0	0	0	0	0	0
North American Neuro-Ophthalmology Society	0	0	0	0	0	0	0	0
North American Spine Society	0	0	0	0	0	0	0	0
Obesity Medicine Association	0	0	0	0	0	0	0	0
Society for Cardiovascular Angiography and Interventions	0	0	0	0	0	0	0	0
Society of American Gastrointestinal Endoscopic Surgeons (e)	0	0	0	0	0	0	0	0
Society of Cardiovascular and Computed Tomography	0	0	0	0	0	0	0	0
Society of Critical Care Medicine	0	0	0	0	0	0	0	0
Society of Interventional Radiology	0	0	0	0	0	0	0	0
Society of Nuclear Medicine and Molecular Imaging	0	0	0	0	0	0	0	0
Society of Thoracic Surgeons	0	0	0	0	0	0	0	0
Spine Intervention Society	0	0	0	0	0	0	0	0
The Society of Laparoscopic and Robotic Surgeons	0	0	0	0	0	0	0	0
The Society of Laparoscopic and Robotic Surgeons	0	0	0	0	0	0	0	0
Undersea and Hyperbaric Medical Society	0	0	0	0	0	0	0	0

Key: (a) includes content regarding climate change (4 vs. 0 points), (b) includes content regarding climate change (2 vs. 0 points), (c) includes content regarding climate change (1 vs. 0 points), (d) member or participating organization (4 vs. 0 points), (e) Some sections password protected

The scores of specialty societies on metrics related to their inclusion of climate change content are shown in Table 1. Based on a maximum obtainable score of 21 points (100%), the total scores for the medical organizations ranged from zero to 19 (0 – 90%). Fifty organizations were found to have at least one metric with a reference to climate change while sixty-one organizations had no evidence of content concerning climate change (Table 1). Five organizations obtained total scores greater than 80%: the American Academy of Family Physicians, the American Academy of Pediatrics, the American College of Physicians, the American Medical Association and the Infectious Disease Society of America.

Position statements or policies pertaining to climate change were found in 22 (20%) of organizations (Table 2). Notable examples of comprehensive and directive policies can be found on the websites of the American College of Obstetricians and Gynecologists, the American Academy of Dermatology and the American Society of Anesthesiologists [27–29]. Committees or task forces dealing with climate change were noted in 12 (11%) websites, with robust examples demonstrated by the American Academy of Allergy Asthma and Immunology and the American College of Radiology [30, 31]. Only 9 (8%) organizations provided patient education resources on climate change, most notably the American College of Physicians [32]. Green practice resources were noted for 23 (21%) organizations, with an extensive list of “Greening the Health Care Sector'' documents on the American College of Physicians website [33]. The most frequently positive metric was the category of climate change articles included in official society publications or blogs, found for 50 (45%) of the organizations. Many organizations referred to materials generated by the Medical Society Consortium on Climate and Health, with a few mentions of My Green Doctor. Fifteen (14%) organizations were listed as member societies of the Medical Society Consortium on Climate and Health website while only 2 (2%) were included as participating organizations on the My Green Doctor website [24, 25].

**Table 2:** Summary of Website Informational Content on Climate Change for National Medical Societies and AMA

Website Informational Content regarding Climate Change	Number of Organizational Websites containing Content No. (%) (*N* = 111)
At least one positive metric on climate change	50 (45)
Position statements or policies	22 (20)
Committee or task force	12 (11)
Patient education	9 (8)
Practice recommendations	23 (21)
Publications or blogs	50 (45)
Member society of Medical Society Consortium on Climate and Health	15 (14)
Participating organization of My Green Doctor	2 (2)

Medical societies addressed two distinct aspects of climate change on their websites: a) adaptation to the health impacts of climate change and b) mitigation of the carbon footprint of medical practices. Most of the highest scoring organizations had a balanced approach, addressing both issues in policy statements as well as scoring positively on the patient education resources and green practice recommendations metrics. Some organizations with a minimal carbon footprint, such as the American Psychiatric Association, appeared to focus on the health impacts of climate change. In contrast, other organizations less involved in direct or ongoing patient care appeared to focus on the reduction of their carbon footprints, such as the American Society of Anesthesiologists and the American College of Radiology.

As an exploratory investigation, the annual meeting formats for 2021 and 2022 were evaluated for the 50 organizations that recorded at least one positive metric (Table 3). The widespread adoption of a virtual attendance option in 2021 was mandated by health concerns related to the COVID-19 pandemic, but it could also be leveraged to reduce organizations’ carbon footprints [26]. In 2021, the format of the annual meeting was available for 47 organizations: 31 (62%) of the meetings were virtual, 15 (30%) were hybrid and only 1 (2%) meeting was held in-person. In 2022, as the pandemic wanes, the formats of the annual meetings for the 49 with available information have shifted: only 1 (2%) has plans for a virtual meeting while 34 (68%) are planning for a hybrid format and 14 (28%) are planning to return to an in-person format. Of note, all of the websites that explained the use of a virtual option related it to the pandemic, while only 1 (2%) referenced the benefit of reducing the carbon footprint of attendees.

**Table 3:** Annual meeting format for organizations with at least one positive metric

Format	2021 No. (%) (*N* = 50)	2022 No. (%) (*N* = 50)
Virtual	31 (62)	1 (2)
Hybrid (virtual + in-person)	15 (30)	34 (68)
In-person	1 (2)	14 (28)
No information	3 (6)	1 (2)

## Discussion

We evaluated the involvement of the U.S. medical community in climate change through the lens of medical society websites, official society journal publications, and national meetings. We found wide variation in climate change advocacy. The climate emergency must be addressed vigorously and expeditiously at all levels of society in order to lessen its impact. The U.S. health care sector has a unique role: as an industry it produces a substantial amount of carbon emissions [8], while its professional workers have an ethical and moral responsibility to protect patients from the adverse impacts of climate change and to educate patients on how they can proactively fight climate change [34]. The U.S. lacks a strong centralized health care authority, such as the United Kingdom’s National Health Service, that can drive the health sector's actions towards mitigation and adaptation [35]. A recent review of U.S. public health department websites revealed that fewer than half (40%) of state health department websites, and only 1.6% and 3.9% of county and city websites, respectively, contained any information for the public about climate change [18]. The U.S. federal government can influence the behavior of the health care sector to some degree [9, 36], but we will need additional robust strategies, including a unified medical community, to enable the generational change required to address the climate crisis.

Our national medical societies need to take a leading role in directing the health sector to mitigate its carbon emissions and guide our patients in adapting to climate change. Medical providers depend on these organizations to provide direction in health care policies and to promote best practices for our medical facilities [37]. Our medical societies are adept in the development and dissemination of physician and patient education materials. The COVID pandemic has underscored the importance of medical society websites, which have provided a forum for rapid dissemination of updated guidelines and recommendations for patient care [17]. Further, physicians remain highly trusted by patients and are well-positioned to discuss issues related to climate change with patients [15]. An international survey of health professionals highlighted the need for continuing professional education and policy statements on climate change and health by their professional associations [16].

Our review demonstrates that about twenty national medical societies are already actively addressing climate change. Mobilization of the remainder of the organizations will require astute leadership and/or a groundswell of support from concerned members. One author (TB) has been involved in an effort to persuade their specialty organization to adopt a more vigorous approach to climate change [38]. Although this review was not set up to evaluate the effectiveness of various measures that organizations can undertake to address the climate crisis, it would be reasonable to presume that a solid foundation would require the crafting of strong position or policy statements and the formation of devoted committees or task forces. The top scoring organizations in our survey all posted position statements and policies that recognized the significant health risks of climate change, and committed to various actions to mitigate and adapt to the crisis.

A relatively small number of organizations posted patient educational materials regarding climate change, while a larger number provided some green practice references or resources. We have highlighted two organizations, the Medical Society Consortium on Climate and Health and My Green Doctor, since they are well positioned to work with medical societies and individual providers to quickly and efficiently address the climate emergency with a wealth of information and resources [24, 25]. Other organizations are doing excellent work in this field and could be consulted – including Climate and Health Alliance, Health Care without Harm, Practice Greenhealth and Planetary Health Alliance [20–23].

Many of the top scoring medical societies represent providers that care for large patient populations, including family practitioners (American Association of Family Practitioners), pediatricians (American Academy of Pediatrics), and internists (American College of Physicians and the American Medical Association). Given the increased risks of vector-borne and other infectious diseases that accompany climate change, it is not surprising that the Infectious Disease Society also scored highly in our survey. These organizations generally exhibited a balanced approach to both educate and protect their vulnerable patients as well as to reduce their carbon footprints.

Procedure and technology rich specialties may have little or no ongoing direct patient care responsibilities, but they are likely to have a large carbon footprint. Examples include greenhouse gas emissions of volatile anesthetics (e.g., halogenated fluorocarbons), and use of energy intensive equipment for medical imaging (e.g., scanners, reading stations). Accordingly, the organizations with higher scores reflecting an emphasis on the reduction of their carbon footprints included specialties such as anesthesia and radiology.

We believe that medical societies should aim to address both (a) the health impacts of climate change and (b) mitigation of their carbon footprint. The scoring system we employed highlighted these goals in the “patient education” and “practice recommendation” metrics but did not specifically capture the weighting of these goals in either the “position statement and policy” metric or the “committee and task force” metric. In future surveys, it would be helpful to differentiate the relative contributions to these domains in order to better acknowledge current progress as well as to identify areas for improvement.

The publications and blogs promoted by national medical associations tended to address climate change issues much more readily than the organizations themselves. The bar for a robust literature on climate change has been set by the Lancet Countdown [39] and the NEJM Group Series [40], with more research urgently needed to comprehend the impact of the climate crisis by all disciplines.

Medical societies adapted to the constraints of the COVID-19 pandemic by leveraging technology to provide for virtual attendance at most annual meetings in 2021. As the pandemic wanes in 2022, most organizations are planning to retain a hybrid virtual option, though many are returning to an in-person format [41]. Employing a virtual option for attendance can reduce the carbon footprint of meetings which average emissions of at least 1.5 metric tons of CO_2_per attendee [42, 43]. The leaders of our medical organizations should leverage this technology as a means to reduce their carbon emissions [44]. The use of a hybrid format provides a mechanism to reduce carbon footprints but still offer in-person attendance when deemed necessary by program planners.

A primary limitation of our study was the lack of available metrics or scoring systems to evaluate publicly facing medical society websites for evidence of involvement in climate change issues. The authors created de novo search parameters and a novel scoring system that we felt best represented the key domains needed for an effective climate change response. A similar approach has been utilized to evaluate health department websites for the inclusion of climate change content [18] and to determine the quality of information presented on websites regarding methotrexate [45]. Another potential limitation is that the cross-sectional snapshot of the visible content of websites may not have fully reflected the current level of organizational engagement in addressing climate change issues. Such organizations should consider adding content to their social media platforms using the model for dissemination of information developed for COVID-19 [17]. Some data may have been inaccessible due to password protected sections of websites as noted in Table 1 and Additional File 1. As review of website content may be subject to errors of omission or interpretation, we mitigated this possibility by creating a structured template for our website searches.

## Conclusions

It is incumbent upon the U.S. health sector to employ every available tactic to reduce our carbon emissions, and to devise effective strategies to educate and protect our patients. Our medical societies need to play a key role in mitigation efforts and adaptation to climate change. Although some organizations are already committed and active in addressing climate change, most need to rapidly improve their efforts: the high scoring organizations in our survey can serve as models for those just beginning this task. We would encourage members of organizations that are demonstrating low scores to demand that their organizations begin the process of assessing the health impacts of climate change on their patient populations and/or addressing the sustainability of their industry and practices.

## Supplementary Information

**Additional file 1.** Deleted National Medical Organizations without a functioning search box.

## Data Availability

All data generated or analyzed during this study are included in this published article [and its supplementary information files.
