# Supplementary material for: U.S. medical organizations and climate change advocacy: a review of public facing websites
Source: BMC Public Health. 2022 Oct 21;22:1950. doi: 10.1186/s12889-022-14339-7 (PMC9585738; doi:10.1186/s12889-022-14339-7)
Supplement: Supplementary file 1 — Additional file 1. Deleted National Medical Organizations without a functioning search box. [file 12889_2022_14339_MOESM1_ESM.docx]

**Additional File 1** – Deleted National Medical Organizations without a functioning search box

| 1 | American Academy of Pain Medicine |
| --- | --- |
| 2 | American Academy of Psychiatry and the Law |
| 3 | American Association for Geriatric Psychiatry |
| 4 | American Association of Clinical Urologists, Inc. |
| 5 | American College of Legal Medicine |
| 6 | American Society for Reconstructive Microsurgery |
| 7 | American Society of Echocardiography |
| 8 | American Society of Neuroimaging |
| 9 | International College of Surgeons: U.S. Section |
| 10 | Society for Investigative Dermatology |
| 11 | The Triological Society |
| 12 | United States and Canadian Academy of Pathology |
